# Differential regulation of caffeine metabolism in *Coffeaarabica* (Arabica) and *Coffea canephora* (Robusta)

**DOI:** 10.1007/s00425-014-2170-7

**Published:** 2014-09-24

**Authors:** Charlène Perrois, Susan R. Strickler, Guillaume Mathieu, Maud Lepelley, Lucie Bedon, Stéphane Michaux, Jwanro Husson, Lukas Mueller, Isabelle Privat

**Affiliations:** 1 Nestlé R&D Center, 101 Av. Gustave Eiffel, Notre Dame D’Oé, BP 49716, 37097 Tours, France; 2 Boyce Thompson Institute for Plant Research, Tower Road, Ithaca, NY 14853 USA

**Keywords:** Arabica, Beans, Caffeine, Expression, Leaves, *N*-methyltransferase, Robusta

## Abstract

Caffeine is a metabolite of great economic importance, especially in coffee, where it influences the sensorial and physiological impacts of the beverage. Caffeine metabolism in the *Coffea* species begins with the degradation of purine nucleotides through three specific *N*-methyltransferases: XMT, MXMT and DXMT. A comparative analysis was performed to clarify the molecular reasons behind differences in caffeine accumulation in two *Coffea* species, namely *Coffea arabica* and *Coffea canephora* var*. robusta*. Three different genes encoding N-methyltransferase were amplified in the doubled haploid *Coffea canephora: CcXMT1*, *CcMXMT1* and *CcDXMT*. Six genes were amplified in the haploid *Coffea arabica*: *CaXMT1*, *CaXMT2*, *CaMXMT1*, *CaMXMT2*, *CaDXMT1,* and *CaDXMT2*. A complete phylogenic analysis was performed to identify specific key amino acids defining enzymatic function for each protein identified. Furthermore, a quantitative gene-expression analysis was conducted on leaves and on maturing coffee beans, simultaneously analyzing caffeine content. In the different varieties analyzed, caffeine accumulation is higher in leaves than in the coffee bean maturation period, higher in Robusta than in Arabica. In Robusta, *CcXMT1* and *CcDXMT* gene expressions are predominant and transcriptional activity is higher in leaves than in maturing beans, and is highly correlated to caffeine accumulation. In Arabica, the *CaXMT1* expression level is high in leaves and *CaDXMT2* as well to a lesser extent, while global transcriptional activity is weak during bean maturation, suggesting that the transcriptional control of caffeine-related genes differs within different organs and between Arabica and Robusta. These findings indicate that caffeine accumulation in *Coffea* species has been modulated by a combination of differential transcriptional regulation and genome evolution.

**Electronic supplementary material:**

The online version of this article (doi:10.1007/s00425-014-2170-7) contains supplementary material, which is available to authorized users.

## Introduction

Coffee is an important crop with more than seven million tons of green beans produced every year. After oil, coffee ranks second in international trade exchanges. The two main species cultivated throughout the tropical world are *Coffea arabica* and *Coffea canephora,* which represent 70 and 30 %, respectively in world production. In terms of cup quality, *Coffea arabica* (Arabica) is appreciated by consumers due to better flavor and high acidity compared with *Coffea canephora* (Robusta), famous for its bitterness and intense dark flavor. Coffee quality, with complex variables, depends significantly on the fluctuating biochemical composition of the bean during fruit maturation (Simkin et al. 2006; Lepelley et al. 2007; Privat et al. 2008; Salmona et al. 2008) as well as control on the genetic level (Montagnon et al. 1998; Leroy et al. 2006). The principal molecules accumulated in coffee beans are caffeine, chlorogenic acids, lipids, sucrose, fat, and proteins. These different aroma precursors are transformed during roasting through Maillard reactions. Typically, chlorogenic acids and caffeine are responsible for coffee bitterness, while free carbohydrates like sucrose generate flavor compounds by interacting with amino acids. Furthermore, sucrose lends sweetness to the final beverage taste. Among the compounds present in coffee beans, caffeine is among the best known and most commonly studied because of its physiological effects on humans and its role in coffee plant resistance.

Caffeine is a clear target for breeding programs to obtain natural low-caffeine-content coffee or on the contrary to increase caffeine content to produce darker and stronger coffee. Caffeine accumulation studies among the *Coffea* species (Ky et al. 2001; Campa et al. 2004, 2005; Ashihara and Suzuki 2004) have highlighted a broad range of diversity from no caffeine in *C. pseudozanguebariae* to the highest content in *Coffea canephora* (Robusta). A segregation study on an interspecific cross between *Coffea liberica* and *Coffee pseudozanguebariae* showed two major QTLs controlling caffeine and chlorogenic-acid content in beans (Ky et al. 2013). Breeding strategies, including QTL identification combined with candidate-gene validation, are highly valuable and may help identify the caffeine-metabolic pathway in *Coffea* species characterized by different levels of caffeine content.

The biosynthetic pathway of caffeine has been intensively studied and it has now been established that caffeine is synthesized through the sequential three-step methylation of xanthosine derivatives at positions 7-N, 3-N and 1-N, with a nucleosidase reaction leading to the synthesis of the following molecules: the 7-methylxanthosine, the 3,7-methylxanthine (theobromine) and last, the 1,3,7-trimethylxanthine (caffeine) (Ashihara and Crozier 1999; Ashihara and Suzuki 2004). The three *N*-methyltransferases were designated as xanthosinemethyltransferase (XMT), 7-methylxanthine transferase (MXMT) and 3,7-dimethylxanthine methyltransferase (DXMT). Numerous studies have been launched aiming to identify the genes involved in caffeine biosynthesis (Ogawa et al. 2001; Uefuji et al. 2003; Mizuno et al. 2003b). The different published sequences exhibit a high degree of sequence similarity (>80 %) with each other. They also show substrate specificity allowing us to define key motifs/amino acids involved in enzymatic activity (Mizuno et al. 2003a; Uefuji et al. 2005; McCarthy et al. 2007). Most of the available sequences have been identified in *Coffea arabica* but before this year, no comparative analysis had been performed to elucidate the molecular reasons for the difference in caffeine accumulation in different *Coffea* species. Our major focus was to perform a comparative analysis at different levels: (1) gene identification and annotation, (2) caffeine quantification and, (3) analysis of the gene expression encoding the different *N*-methyltransferases in *Coffea* species characterized by varying caffeine contents. The main objective is to propose a first explanation for the differences in caffeine accumulation in coffee.

## Materials and methods

Plant material fresh cherries were harvested from coffee trees cultivated in Quito, Ecuador, at different stages of development [small green fruit (SG), large green fruit (LG), yellow fruit (Y) and red fruit (R)] (Privat et al. 2008). For each maturation stage, only 15–20 coffee cherries were available for each genotype analyzed. Therefore, performing three biological replicates on the same genotype was not possible. Thus, to draw conclusions for each species, several genotypes were used that were considered as biological replicates. Four genotypes were used for this study: *Coffea arabica* L*. cv. Caturra* (Arabica) CCCA12 and CCCA02, and *Coffea canephora* var. *robusta* (Robusta) FRT05 and FRT64. Due to the fact that Robusta cherries develop over a period of 9–11 months while Arabica fruits develop over a 6–8 month period (Wintgens 2004), the ripening stages were classified by the relative parameters of size, weight and color change, rather than by weeks after flowering (Bargel and Neinhuis 2005). Fresh tissues were frozen immediately and then packaged in dry ice or frozen to −25 °C for transportation, then stored at −80 °C until use. The bean and pericarp tissues were separated for each stage of maturation. Young and mature leaves were collected from different genotypes grown under greenhouse conditions in Tours, France: *Coffea arabica* L*. cv. Caturra* (Arabica) CCCA12, CCCA02, CCCA24, CCCA18; *Coffea canephora* var. *robusta* (Robusta) FRT05, FRT64. Last, leaves from the *Coffea canephora* accession DH-200-94 and the *Coffea arabica* ET39-DH3 were collected to characterize the genes or cDNA sequences encoding the caffeine-synthesis-related genes. Frozen tissues were then homogenized using a SPEX CertiPrep 6800 freezer mill with liquid nitrogen. These different samples were used for RNA, genomic extraction and/or biochemical analysis as previously (Lepelley et al. 2007; Privat et al. 2008).

### Extraction of total RNA and cDNA preparation

Samples stored at −80 °C were ground into a powder and total RNA was extracted from this powder using the method described previously (Rogers et al. 1999). Samples were treated with DNase using the “Qiagen RNase-Free DNase” kit in accordance with the manufacturer’s instructions on removing DNA contamination. All RNA samples were analyzed by formaldehyde agarose gel electrophoresis and by visual inspection of the ribosomal RNA bands on ethidium bromide staining. Furthermore, the concentration was determined on a Nanodrop spectrophotometer (NanoDrop Technologies). Using oligo (dT_20_) as a primer, cDNA was prepared from 1 µg total RNA, according to the protocol in the superscript III reverse transcriptase kit (Invitrogen, Carlsbad, CA, USA). The first-strand cDNA synthesis incubation step was performed for 50 min at 50 °C. The cDNA samples were then diluted 100-fold in sterilized water and stored at −20 °C for later use in a quantitative real-time polymerase chain-reaction (q-RT-PCR) analysis or cDNA full-length amplification.

### cDNA and genomic amplification for caffeine-related gene characterization

To amplify full-length cDNA or genomic sequences encoding the different *N*-methyltransferases involved in caffeine synthesis, the following primer sets—CAF1-ATG (^5′^ATGGAGCTCCAAGAAGTCCTGCA^3′^), CAF1-STOP (^5′^TTACACGTCTGACTTCTCTGGCT^3′^) and CAF2-ATG (^5′^ATGGAGCTCCAAGAAGTCCTGCG^3′^), CAF2-STOP (^5′^TTACATGTCTGACTTCTCTGGCT^3′^) were designed on the consensus sequence obtained from the alignment of the different N-methyltransferases available in the public databases. These two primer sets were used to perform PCR reactions using cDNA or genomic DNA samples prepared from *Coffea canephora* accession DH-200-94 and the *Coffea arabica* ET39-DH3. The PCR reactions were performed in 50 µL reactions as follows: 5 µL of cDNA or gDNA; 1× buffer, 800 nM of each gene-specific primer, 200 µM of each dNTP, and 0.5 U of LA Taq polymerase (Invitrogen). After denaturing at 94 °C for 5 min, the amplification consisted of 35 cycles of 1 min at 94 °C, 1 min at 55 °C and 2 min at 72 °C. An additional final step of elongation was carried out at 72 °C for 7 min. Fragments obtained were purified from agarose gel, cloned and sequenced. To prevent any errors from PCR amplification, each PCR was performed in duplicate, the fragments being cloned separately from two independent ligations.

### DNA Sequencing and sequences analysis

For DNA sequencing, recombinant plasmid DNA was prepared and then sequenced by GATC (Konstanz, Germany). Computer analysis was performed using DNA Star (Lasergene, DNAstar Inc., Madison, WI, USA) software. Sequence homologies were verified against GenBank databases using BLAST programs (Altschul et al. 1990). The sequence encoding each gene was validated whenever the same sequence was obtained from two independent PCRs. Each sequence identified in this article was submitted to the NCBI database (Table 1).

**Table 1:** Identification of the genes encoding the *N*-methyltransferase involved in caffeine synthesis in *Coffea arabica* and *Coffea canephora* species

Species	Gene name	Genomic sequence		mRNA sequence	
*Coffea canephora* DH-200-94	*CcXMT1*	JX978509	1994 pb	JX978518	1119 b
	*CcMXMT1*	JX978507	1829 pb	JX978517	1137 b
	*CcDXMT*	JX978506	2006 pb	JX978516	1155 b
*Coffea arabica* ET39-DH3	*CaXMT1*	JX978514	1987 pb	JX978521	1119 b
	*CaXMT2*	JX978515	2038 pb	JX978522	1158 b
	*CaMXMT1*	JX978511	1838 pb	JX978519	1137 b
	*CaMXMT2*	JX978512	2010 pb	JX978520	1155 b
	*CaDXMT1*	JX978510	2063 pb	KF678863	1155 b
	*CaDXMT2*	KJ577792	2006 pb	KJ577793	1155 b

For each species, the amplified genomic sequences as well as the corresponding mRNA have been annotated. For each sequence, the accession number in NCBI is specified as well as the sequence length

### Quantitative real time PCR

Alignment of the genomic sequences encoding the caffeine-related genes (Supplementary Fig. S1) shows a high similarity between the different sequences identified. The Exon 3 region shows the highest level of polymorphism within the different genes, leading us to design all the primers/TaqMan probe pairs in this region. For each gene, primers/probe pairs were designed on the different sequences available using primer express software (Applied Biosystems, Foster City, CA, USA) (Supplementary Table S1). The TaqMan PCR reactions were carried out according to the manufacturer’s instructions (Applied Biosystems). For all primers/probe sets, standard curves were generated using serial dilutions of plasmid DNA containing the appropriate target-gene sequence. Ct values were determined and plotted versus the natural logarithm of the DNA concentration. Regression analysis provided a linear function from which the PCR efficiency could be calculated using the equation *E* = *e* ^*−*1/*m*^ − 1, where *E* is the PCR efficiency, *e* Euler’s number and *m* the slope of the regression function. Efficiency values were determined for each gene. Furthermore, to prevent any unspecific amplification, the primer/probe designs for one specific gene were tested on the different sequences identified in this publication. Only the primers/probe pairs that showed no unspecific amplification were used for further analysis. The cDNA samples used in this experiment have been described above. All reactions contained 1× TaqMan buffer (Applied Biosystems) and 5 mM MgCl_2_, 200 µM each of dATP, dCTP, dGTP and dTTP, 100-fold dilution of cDNA corresponding to 0.001 μg of original RNA and 0.625 units of AmpliTaq Gold polymerase. A PCR was carried out using 800 nM of each gene-specific primer, forward and reverse, and 200 nM of the corresponding TaqMan probe. Reaction mixtures were incubated for 2 min at 50 °C, 10 min at 95 °C, followed by 40 amplification cycles of 15 s at 95 °C/1 min at 60 °C. Samples were quantified in the GeneAmp 7500 Sequence Detection System (Applied Biosystems). Transcript levels were normalized using *rpl39* (large ribosomal subunit 39) and *UBQ* (ubiquitin-like protein) as reference genes (Cruz et al. 2009; Lepelley et al. 2012). The values represent the mean of three technical repetitions ± standard deviation.

### Caffeine quantification

The coffee material was ground with liquid nitrogen, sifted through a 500 μm sieve, and immediately stored at −20 °C until use. The ground coffee material (10 mg) was extracted in de-ionized water containing 70 % methanol. The resulting mixture was macerated by stirring for 30 min at 40 °C. The mixture was then filtered using GHP Acrodisc 0.2 µm filter. The alkaloids and chlorogenic acids were analyzed by HPLC (U3000 from Dionex) on an ACE RP18 (250 × 4 mm, 5 µm) column. The injected sample volume was 10 µL with a flow rate of 0.8 mL/min. The caffeine content is expressed in percentage of dry weight (% DW).

### Bioinformatics and phylogenic analysis

Protein alignments were generated using Clustal W (Thompson et al. 1994) with default values as implemented in Mega v. 5.2 (Tamura et al. 2011). A visual inspection of the alignment was conducted to ensure optimal results. Phylogenetic analysis was performed using 371 amino acid sites including gaps and 19 sequences. A maximum likelihood tree was generated with Mega v. 5.2 (Tamura et al. 2011) using a Poisson substitution model and gamma-rate distribution among sites. Nearest-neighbor-interchange was used as the heuristic tree search method with 100 bootstrap samples. Jalview v. 2.0 (Waterhouse et al. 2009) was implemented to produce an alignment image for publication. A tree image for publication was generated using FigTree v 1.4 (http://tree.bio.ed.ac.uk/software/figtree/). To find putative amino acids conferring functional specificity to each of the three *N*-methyltransferase clusters, the alignment was imported into multi-Harmony (Brandt et al. 2010) along with the protein structure of the *N*-methyltransferase 2EG5E from *C. canephora* as a reference.

## Results

### Isolation and characterization of caffeine metabolism-related genes

**Fig. 1:**
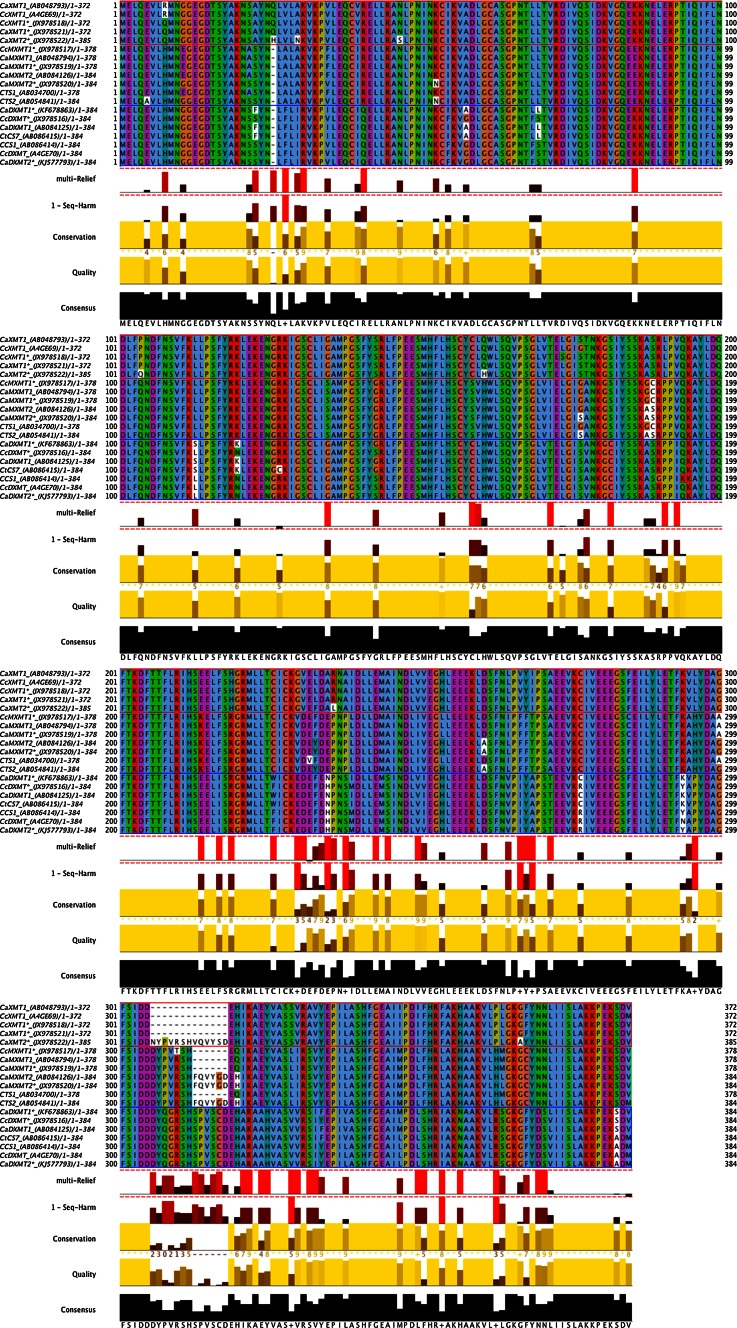
Multiple sequence alignment of nineteen *N*-methyltransferases involved in caffeine metabolism with multi-Harmony results. The Seq-Harm track displays the Sequence Harmony score for each site ranging from 0 to 1. The Multi-relief track shows the weight for each site. GenBank accession numbers are as follows: CaXMT1 (AB048793); CcXMT1 (A4GE69); CcXMT1* (JX978518); CaXMT1* (JX978521); CaXMT2* (JX978522); CcMXMT1* (JX978517); CaMXMT1 (AB048794); CaMXMT1* (JX978519); CaMXMT2 (AB084126); CaMXMT2* (JX978520); CTS1 (AB034700); CTS2 (AB054841); CaDXMT1* (KF678863); CcDXMT* (JX978516); CaDXMT1 (AB084125); CtCS7 (AB086415); CCS1 (AB086414); CcDXMT (A4GE70); CaDXMT2* (KJ577793). The sequences marked with an *asterisk* have been identified in this article

**Fig. 2:**
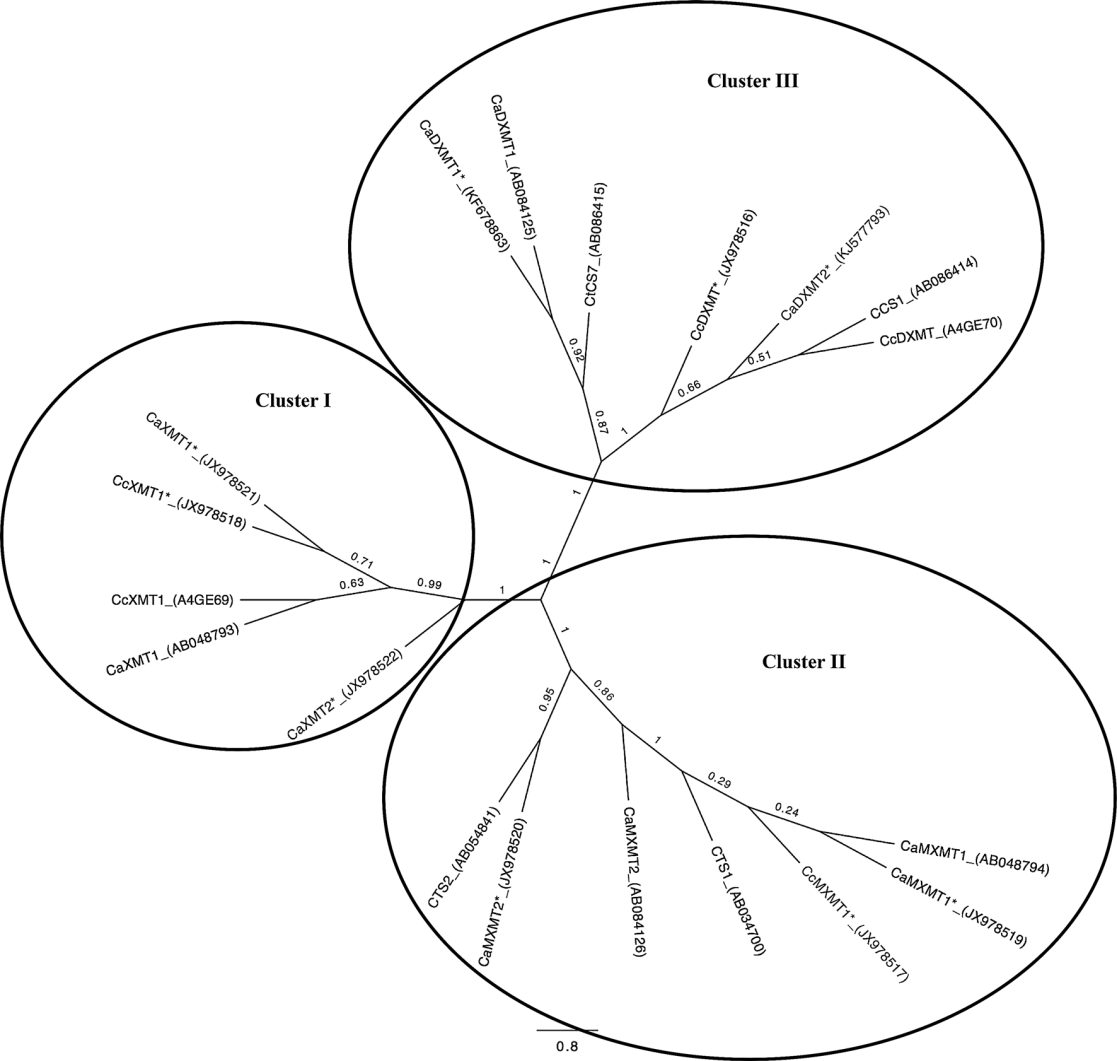
Unrooted maximum likelihood tree based on the alignment of 19 *N*-methyltransferases involved in caffeine metabolism. GenBank accession numbers are as follows: CaXMT1 (AB048793); CcXMT1 (A4GE69); CcXMT1* (JX978518); CaXMT1* (JX978521); CaXMT2* (JX978522); CcMXMT1* (JX978517); CaMXMT1 (AB048794); CaMXMT1* (JX978519); CaMXMT2 (AB084126); CaMXMT2* (JX978520); CTS1 (AB034700); CTS2 (AB054841); CaDXMT1* (KF678863); CcDXMT* (JX978516); CaDXMT1 (AB084125); CtCS7 (AB086415); CCS1 (AB086414); CcDXMT (A4GE70); CaDXMT2* (KJ577793). The sequences marked with an *asterisk* have been identified in this article. Clusters I, II and III correspond to XMT, MXMT and DXMT protein, respectively

To identify the different genes involved in the final steps of the caffeine biosynthesis pathway in two *Coffea* species characterized by different levels of caffeine content, we decided to make use of the fact that previous sequences shared high similarity (Ogawa et al. 2001; Uefuji et al. 2003; Mizuno et al. 2003b; McCarthy and McCarthy 2007) and to design “universal” caffeine gene primers (see Materials and methods). To eliminate the natural polymorphism/allelism existing in *Coffea* species that could interefere with sequence annotation, we selected two specific *Coffeas*: the doubled-haploid *Coffea canephora* Robusta DH-200-94, diploïd but strictly homozygous, and the haploid *Coffea arabica* ET39-DH3 which has only one copy of each subgenome. For the two species, the different genomic sequences characterized are listed in Table 1. The cDNA sequences were amplified from mRNA extracted from leaf samples. When possible, the genomic and mRNA sequences were aligned, successfully indicating the intron/exon junction and identifying the correlation between genomic and mRNA sequences. The alignment between all the genomic sequences encoding *N*-methyltransferases which are involved in caffeine metabolism shows a similar organization (Supplementary Fig. S1). The genomic sequence length varies from 1,829 to 2,063 bp. Each gene has four exons and three introns. Three different genes encoding *N*-methytransferase were amplified in the doubled haploid *Coffea canephora CcXMT1* (JX978509), *CcMXMT1* (JX978507), *CcDXMT* (JX978506). Six genes were amplified in the haploid genome of *Coffea arabica,* i.e., *CaXMT1* (JX978514), *CaXMT2* (JX978515), *CaMXMT1* (JX978511), *CaMXMT2* (JX978512), *CaDXMT1* (JX978510) and *CaDXMT2* (KJ577792). Interestingly, for each enzyme, two paralogue genes were identified in the *Coffea arabica* genome (Table 1). For each gene, the corresponding cDNA was amplified using RNA extracted from leaves (Table 1). The highest variability between the coding sequences is clearly localized in Exon 4 (Supplementary Fig. S1), where specific gaps in the sequence differentiate XMT1 from the MXMT1 genes in Arabica and Robusta. Furthermore, variability, especially in the Intron 2 and Intron 3 regions, helped to identify the specific orthologues between the two *Coffea* species. The protein sequences deduced from each identified gene were aligned (Fig. 1). A phylogenic analysis was performed including the 9 proteins identified in this article as well as various *N*-methyltransferases previously published for different Arabica and Robusta genotypes (Fig. 2). This phylogenic analysis indicated that the different *N*-methyltransferases involved in caffeine synthesis belong to 3 different clusters which align with the function of each protein. Indeed, Clusters I, II and III correspond respectively to XMT, MXMT and DXMT. The different proteins were shown to share more than 80 % similarity throughout the two *Coffea* species. It is interesting to note that within Clusters I, II and III, the percentage of similarity is between 94 and 100 %. The first cluster includes the different genes encoding the XMT enzyme. The two genes *CaXMT1* (JX978521) and *CcXMT1* (JX978518) were identified (Fig. 2) and are closely related to proteins published in the databases CaXMT1 (AB048793) (Ogawa et al. 2001) and CcXMT1 (A4GE69) (McCarthy and McCarthy 2007; McCarthy et al. 2007). An additional gene, *CaXMT2* (JX978522), was identified in the haploid *Coffea arabica* that has no closely related gene in the *Coffea canephora* genome. These results suggest that in *Coffea arabica*, the genes *CaXMT1* and *CaXMT2* are, respectively encoded by the Robusta and Eugenioides sub-genomes. The second cluster includes the genes encoding the MXMT enzyme. The genes *CaMXMT1* (JX978519) and *CcMXMT1* (JX978517) encode the same protein, homologous to the *CaMXMT1* (AB048794) (Ogawa et al. 2001). As it has already been observed for *CaXMT2*, a second gene *CaMXMT2* (JX978520) was also identified (Table 1; Fig. 2); it is clearly the same protein as CTS2 (AB054841) (Mizuno et al. 2003b), also identified in *Coffea arabica*. This gene has no homologue in *Coffea canephora*. In addition, MXMT1 and MXMT2 proteins share 94 % similarity. The third cluster includes the genes encoding the DXMT enzyme. In *Coffea arabica*, two genes, *CaDXMT1* (KF678863) and *CaDXMT2* (KJ577793), have been identified while in *Coffea canephora,* only one gene, *CcDXMT* (JX978516), was identified. Their predicted amino acids share 94 % identity to each other. The members of Cluster III (Fig. 2) define two sub-clusters depending on the origin of the sequence (Arabica or Robusta), suggesting variability among the protein sequences according to the two *Coffea* species analyzed. The proteins CaDXMT2 and CcDXMT are highly homologous and correspond to the protein CcDXMT (A4GE70) and CCS1 (AB086414) (Mizuno et al. 2003a) previously identified. CaDXMT1 (KF678863) corresponds to CaDXMT1 (AB084125) (Uefuji et al. 2003) and CtCS7 (Uefuji et al. 2003). CaDXMT2 and CaDXMT1 proteins are, respectively encoded by the Robusta and Eugenioides sub-genomes in *Coffea arabica* genome. To find putative amino acids conferring functional specificity to each of the three clusters, the alignment (Fig. 1) was imported into Multi-Harmony software (Brandt et al. 2010) along with the protein structure of *C. canephora* XMT (2EG5E as a reference). The result of this analysis has highlighted 16 amino acids capable of defining the signature for the members of each cluster (Table 2). Three amino acids are unique for one of the defining clusters. Identifying these residues significantly clarifies the different protein characteristics and will aid in annotating future N-methyltransferases in *Coffea* species.

**Table 2:** Amino acids that support the presence of the four clusters derived from the phylogenetic analysis (see Fig. 1) and multi-Harmony data mining (see Fig. 2)

Amino acids in				
Position^a^		Cluster I XMT	Cluster II MXMT	Cluster III DXMT
31	K		K	R
159	C		S	C
191	L		P	P
193	V		V	I
217	F		F	I
*230*	*G* ^*b*^		*V*	*E*
235	A		E	H/N
*238*	*A*		*P*	*S*
245	A		A	S
268	Y		F	Y
*296*	*L*		*H*	*P*
316	S		L	V
341	F		L	I
344	H		H	N
350	P		H	R
357	N		N	D

^a ^Positions according to the CaXMT1 (JX978518) protein sequence

^b^ The amino acids which are highly indicative for the three clusters are italicized. These amino acids are unique for one of the defined clusters

### Caffeine and theobromine accumulation

**Fig. 3:**
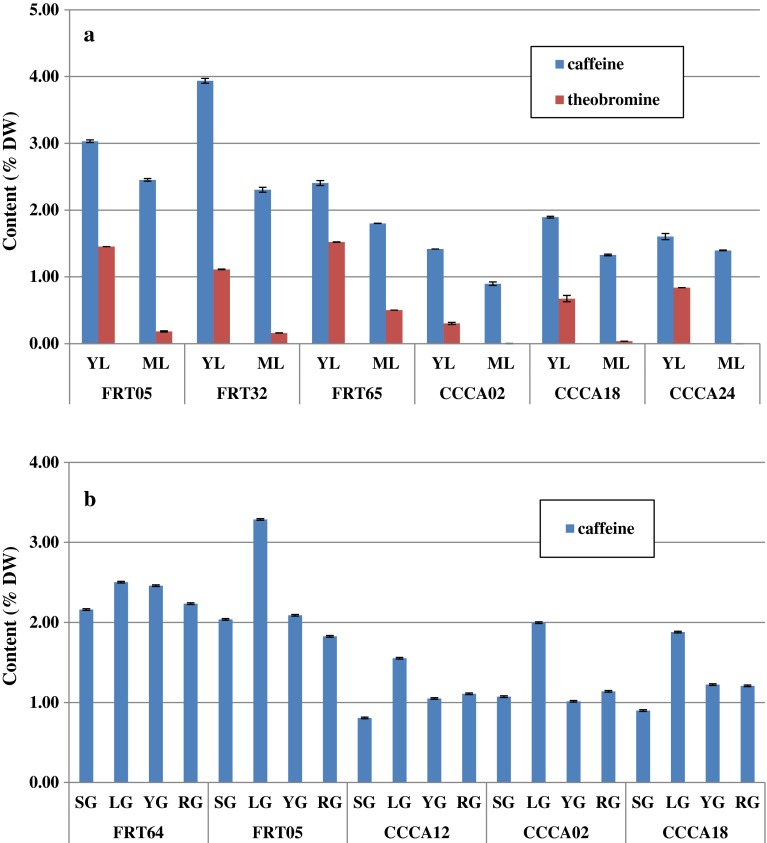
Caffeine and theobromine content during leaf or grain development in different *Coffea* species. **a** Caffeine and theobromine were quantified during leaf development in young (YL) and mature leaves (ML) for different *Coffea arabica* genotypes (CCCA 02, CCCA18, CCCA24), *Coffea canephora* var. *robusta* genotypes (FRT05, FR32, FRT65). **b** Caffeine and theobromine were quantified during coffee bean maturation for different *Coffea arabica* genotypes (CCCA 02, CCCA18, CCCA24), *Coffea canephora* var. *robusta* genotypes (FRT64 and FRT05). The four maturation stages are: *SG* small green grain, *LG* large green grain, *YG* yellow grain, *RG* red grain. The content is expressed in percentage of dry weight (% DW). Values are the mean of three repetitions ± standard deviation

Biochemical analysis indicated an accumulation of theobromine and caffeine in the leaves at both stages in the *C. canephora* and *C. arabica* genotypes (Fig. 3a). Young leaves contain the highest concentration of caffeine in Robusta and Arabica genotypes. On average, Robusta accumulates 3 % (expressed as the percentage of dry weight) and Arabica 1.6 % (DW) of caffeine in young leaves. For both species, in mature leaves, caffeine content drops to 70 % of the initial quantified caffeine content in young leaves. Theobromine is accumulated less in young leaves than caffeine in both species. In young leaves, Robusta accumulates 1.4 % (DW) of theobromine, while Arabica accumulates 50 % less. The reduction of theobromine in mature leaves is particularly apparent for both species, especially for Arabica, where theobromine is no longer detected at all in mature leaves. Interestingly, theobromine is not detected in the beans of Arabica or Robusta during the maturation process (Fig. 3b). In the same samples, caffeine is clearly accumulated in both species, but its highest content is found in Robusta (twice as much) starting even at the earliest stage (SG). Nevertheless, the accumulation profile is the same for Arabica and Robusta. During the expansion phase (from SG to LG stages), caffeine is accumulated even more significantly for Arabica. From LG to YG stages, caffeine content decreases and stabilizes during the last two stages of maturation (YG and RG). In the mature bean (RG stage), the caffeine content is 50 % higher in Robusta than in Arabica. Surprisingly, comparing caffeine accumulation in leaves and beans reveals that caffeine biosynthesis is highest in young leaves.

### Gene expression during leaf and fruit development

**Fig. 4:**
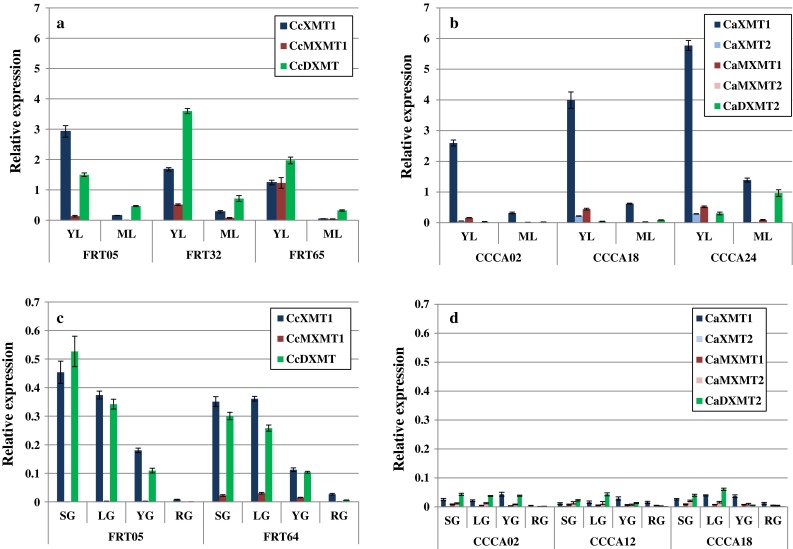
Caffeine biosynthesis enzyme gene expression during leaf or grain development in *Coffea arabica* and *Coffea canephora* (Robusta), determined by q-RT-PCR. Transcript levels were analyzed during leaf development in young (YL) and mature leaves (ML) for different genotypes of *Coffea canephora* var. *robusta* (FRT05, FR32, FRT65, **a**) and *Coffea arabica* (CCCA 02, CCCA18, CCCA24, **b**). Transcript levels were analyzed during coffee bean maturation of different genotypes of *Coffea canephora* var. *robusta* (FRT64 and FRT05, **c**) and *Coffea arabica* (CCCA 02, CCCA12, CCCA18, **d**). The four maturation stages are: *SG* small green grain, *LG* large green grain, *YG* yellow grain, *RG* red grain. The expression levels are determined by quantitative RT-PCR relative to the expression of the constitutively expressed *rpl39* gene in the same samples. In each case, values are the mean of three estimations ± standard deviation. The same code color is used to characterize one gene in Robusta and its homologue in Arabica

Gene expression analysis was performed on leaf and coffee bean development to identify the major differences in the expression pattern of the different genes involved in the final steps of caffeine metabolism. As the different genes are over 80 % identical to each other at the nucleotide level, we decided to conduct a gene expression analysis using quantitative RT-PCR using a TaqMan probe. This technology allowed us to design highly specific probes/primers for each gene. The design was carried out in the most variable region of the coding sequence, i.e., Exon 4 (Supplementary Fig. S1). The gene expression pattern is clearly different between Arabica and Robusta, with drastic divergence in young leaves (Fig. 4a, b). *CcXMT1* and *CcDXMT* are highly expressed in the different Robusta genotypes, *CcDXMT* being higher than *CcXMT1*. *CcMXMT1* is expressed at a lower level in FRT05 and FRT32 genotypes, while it is significantly expressed in FRT65. In the different Arabica genotypes, *CaXMT1* transcripts show high accumulations in young leaves compared to what we observe in Robusta. The highest accumulation is observed in the CCCA24 genotype. *CaXMT2,* the second gene encoding XMT enzyme in Arabica is slightly expressed compared to *CaXMT1* and detected only in young leaves. *CaMXMT1* is detected also only in young leaves at low level compared to *CaXMT1* for example. *CaMXMT1* and *CcMXMT1* are expressed similarly in young leaves while both genes are undetectable in mature leaves. *CaMXMT2* is expressed at very low level in young and mature leaves. The most remarkable difference in Arabica is that *CaDXMT2* is almost undetectable in all the Arabica genotypes in young leaves while the corresponding gene *CcDXMT* in Robusta is highly expressed in young leaves. In both Arabica and Robusta genotypes, the gene expression pattern is modified in mature leaves. Overall gene expression is largely reduced; *CcXMT1* and *CaXMT1* are still detectable, at a higher level for *CaXMT1* in the Arabica genotypes. On the contrary, while *CcDXMT* is still detected in the mature leaves in the Robusta genotypes, *CaDXMT2* is barely detected in the different Arabica genotypes.

During coffee bean development, the expression profile is also quite different between the Arabica and Robusta genotypes (Fig. 4c, d). Overall gene expression is strongly reduced during bean maturation. When a specific transcript is detected, it is accumulated approximately 10 times less than during leaf development in Robusta and 70 times less in Arabica. In Robusta genotypes, as it has already been observed in the young leaf, *CcXMT1* and *CcDXMT* are highly expressed at the same level in the bean expansion phase (SG and LG). Expression decreased drastically in the last stages of maturation, becoming almost undetectable in the RG stage. *CcMXMT1* is only slightly expressed at all stages. For the different Arabica genotypes, all the detected genes (*CcXMT1*, *CaMXMT1*, *CaMXMT2* and *CaDXMT2*) are accumulated at very low levels. *CaDXMT2* accumulation is only slightly higher in the SG and LG stages.

## Discussion

The sequences of the different N-methyltransferase genes involved in the biosynthesis of caffeine were previously identified in coffee and other plant species (Ashihara et al. 2008). However, the fact that these enzymes share highly conserved domains makes it difficult to identify gene-specific sequences for each methyltransferase. Also, the previously published sequences were identified in *Coffea arabica*, allotetraploid species (2*n* = 4*x* = 44 chromosomes) which result from the natural hybridization of two diploid species: *C. canephora* (Robusta) and *C. eugenioides* (Lashermes et al. 1999). The inter- and intra-polymorphism observed between the two sub-genomes greatly hinders the process of identifying the different genes encoding the *N*-methyltransferases. To eliminate any natural polymorphism/allelism existing in the *Coffea* species that could interfere with sequence annotation, specific coffee plants were selected. First, the double-haploid *Coffea canephora* Robusta DH-200-94 was chosen, which is strictly homozygous for each locus. Each polymorphism corresponds to a different sequence and consequently to a different gene. Second, the haploid *Coffea arabica* ET39-DH3, with only one copy of each sub-genome, was used to characterize the genes for Arabica. This strategy was highly successful since the complete set of genes involved in caffeine biosynthesis was amplified in Robusta and Arabica. In Robusta, three genes were identified: *CcXMT1* (JX978509), *CcMXMT1* (JX978507) and *CcDXMT* (JX978506). In Arabica, six genes were identified: *CaXMT1* (JX978514), *CaXMT2* (JX978515), *CaMXMT1* (JX978511), *CaMXMT2* (JX978512), *CaDXMT1* (KF678863) and *CaDXMT2* (KJ577793) (Table 1). It is interesting to note that, based on the phylogenic analysis (Fig. 2), the different orthologues to *CcXMT1*, *CcMXMT1* and *CcDXMT* were clearly identified in *C. arabica* as *CaXMT1*, *CaMXMT1* and *CaDXMT2*, respectively. This analysis suggests that these three genes are encoded by the sub-genome *robusta* in the *Coffea arabica* genome. Consequently, the additional genes *CaXMT2*, *CaMXMT2*, *CaDXMT1* identified in *Coffea arabica* are certainly encoded by the sub-genome *eugenioides*.

These results are highly valuable since it is now possible to assign a specific gene to each sub-genome in Arabica, and thus to determine their respective importance for caffeine accumulation. This analysis gives a global overview of the different genes involved in the three final steps of caffeine synthesis in Arabica and Robusta species. The phylogenic analysis performed on the different sequences identified in the two *Coffea* species indicated that the encoded proteins are organized in three clusters, i.e., XMT, MXMT and DXMT (Fig. 2). Within the entire protein sequence, there are 16 key amino acids that are highly informative for defining the different clusters (Table 2) and their corresponding enzymatic activities. This in-depth analysis lends considerable support to the previous results, showing key amino acids that define enzymatic activity (Ogawa et al. 2001; McCarthy and McCarthy 2007). For example, the role of S_316_ was shown to be crucial for xanthosine substrate specificity in XMT and A_238_ helped distinguish MXMT from DXMT (McCarthy and McCarthy 2007). In the perspective of acquiring coffee plants with reduced caffeine content, these different amino acids would be an interesting target for mutation using TILLING (Till et al. 2003; Kurowska et al. 2011) or TALEN (Beurdeley et al. 2013; Zhang et al. 2013) technology. After using the strategy of “universal primers” to identify the different genes encoding the *N*-methytransferase, surprisingly few additional genes were also amplified in Arabica and Robusta which encode MTL proteins, namely *N*-methyltransferase-like proteins (Uefuji et al. 2003). Previous results have shown that MTL protein are highly homologous with XMT, MXMT and DXMT enzymes, but do not participate in caffeine biosynthesis (Ogawa et al. 2001). A new phylogenic analysis was performed including the different MTL-encoding sequences identified in this work (Supplementary Fig. S2). The MTL proteins are specifically grouped in Cluster IV suggesting a stronger homology within the MTL proteins than with XMT, MXMT or DXMT proteins.

Caffeine metabolism in the *Coffea* species is set off by the degradation of purine nucleotides. Numerous publications investigated caffeine biosynthesis using radio-labeled tracers, metabolite profiling and enzymatic activities for leaf and fruit development (Suzuki and Waller 1984; Fujimori and Ashihara 1994; Zheng and Ashihara 2004; Campa et al. 2004; Koshiro et al. 2006). Active synthesis was detected in young leaves, bean and pericarp (Ashihara et al. 1996; Ashihara and Crozier 1999; De Castro and Marraccini 2006). In the present work, a large-scale analysis was performed to study the correlation between caffeine metabolism and gene expression during leaf and grain development in two *Coffea* species characterized by different caffeine accumulations. Previous gene expression analyses were performed by RT-PCR or classical Northern blot (Ogawa et al. 2001; Uefuji et al. 2003; Mizuno et al. 2003a, b). The high homology between the different sequences encoding the *N*-methyltransferases involved in caffeine metabolism reinforced our idea of screening for gene expression using quantitative RT-PCR, based on Taq-Man probe technology for ensuring the highest specificity and sensitivity for gene detection. To prevent any unspecific amplification, the primer/probe design for one specific gene was tested on the different sequences identified in this publication including MTL encoding genes. Only the primers/probe pairs that showed no unspecific amplification were used for further analysis. Unfortunately, none of the primers/probe pairs designed to analyze *CaDXMT1* gene expression were specific or showed systematically cross hybridization with MTL encoding genes. Thusly, *CaDXMT1* gene expression analysis was not thus analyzed using this technology. The second point to consider in q-RT-PCR analysis is the selection of accurate reference genes to be used for internal control for reliable data normalization. In keeping with previous data published in *Coffea* species, *rpl39* (large ribosomal subunit 39) and *UBQ* (ubiquitin-like protein) were used as reference gene (Privat et al. 2008; Salmona et al. 2008; Cruz et al. 2009; Lepelley et al. 2012). Similar results were obtained using both genes for internal control, suggesting a stable expression level throughout our experimental design.

For the first time, a global analysis was performed on leaf and grain throughout the development in various Robusta genotypes. Our data demonstrate that caffeine metabolism is highly active in young leaves (Fig. 3a) in Robusta, with caffeine levels reaching beyond 2 % (DW). It is interesting to detect that caffeine accumulation seems to be highly dependant on *CcDXMT* expression while theobromine is more closely related to the high accumulation of *CcXMT1* transcripts and to a lesser extend to *CcMXMT1* since its expression is detected at a very low level in the three genotypes studied (Fig. 4a). During leaf development, theobromine content is drastically reduced, which is largely correlated to a lower *CcXMT1* transcripts accumulation. Caffeine is also less accumulated in mature leaves, which correlates to the reduction of *CcDXMT* transcripts accumulation. Surprisingly, caffeine metabolism is lower in beans than during leaf development (Fig. 3a, b). These results are consistent with those obtained previously in leaves (Ashihara et al. 1996; Ashihara and Crozier 1999) or during grain development (Suzuki and Waller 1984; Ashihara and Suzuki 2004; Koshiro et al. 2006). Global transcriptional activity for the genes involved in caffeine metabolism is sharply reduced during bean development (10–20 times less) (Fig. 4a, c). Although, the gene expression pattern in Robusta is the same between endosperm and young leaves, transcript accumulation is significantly reduced for *CcXMT1* and *CcDXMT* genes in the endosperm. *CcXMT1* and *CcDXMT* transcripts are co-expressed, as it was observed in young leaves, with a decrease in accumulation during the last two stages of maturation. Despite the low accumulation of *CcMXMT1* transcripts, the caffeine biosynthesis pathway is complete during bean maturation since the DXMT enzyme can methylate the 7-Methylxanthine and theobromine (Uefuji et al. 2003; McCarthy and McCarthy 2007).

In parallel, the same analysis was performed on leaf and grain in different arabica genotypes revealing great differences in caffeine metabolism depending on the organ and the species analyzed. In the different Arabica genotypes analyzed, caffeine and theobromine are less accumulated than in Robusta in leaves (Fig. 3a, b). At the same time, the gene expression pattern is markedly different, with a very high level of *CaXMT1* expression, higher than its homologue in Robusta. *CaXMT2* is over 10 times less expressed than *CaXMT1* suggesting that *CaXMT1* and *CaXMT2* are controlled differentially at the transcriptional level. *CaMXMT1* is less expressed than *CcMXMT1*; *CaMXMT2* is almost undectectable in the different leaf tissues analyzed (Fig. 4b). These results strongly suggest that XMT1 and MXMT1 in both species drive theobromine accumulation, especially in young leaves. DXMT transcripts accumulation is remarkably different in Arabica. While *CcDXMT* is strongly expressed and directly correlated with caffeine accumulation in leaves, *CaDXMT2* transcripts are poorly accumulated partially explaining the reduced caffeine content in Arabica compared to Robusta. It is interesting to note that in *Coffea arabica*, by analyzing the genes involved in caffeine accumulation, the co-existence of the two sub-genomes has a clear impact on caffeine accumulation. The genes *CaXMT1*, *CaMXMT1* and *CaDXMT2* encoded by the Robusta sub-genome are expressed differently than their homologue in Robusta, drastically reducing caffeine accumulation. Secondly, the genes *CaXMT2*, *CaMXMT2* and probably *CaDXMT1* encoded by the sub-genome Eugenioides are expressed at very low level in Arabica as they certainly are in *Coffea eugenioides,* explaining the low caffeine metabolism (Ashihara and Crozier 1999; Campa et al. 2004; Ashihara 2006) in leaves (Fig. 4a, b). Nevertheless, the assumption made for *CaDXMT1* transcript accumulation would need to be confirmed using RNA-seq technology, for example. With its single-base pair resolution, sensitivity, and replicability, this technology will be highly helpful in distinguishing *N*-methyltransferase involved in caffeine metabolism and in determining the transcript accumulation in different organs. Gene expression for caffeine metabolism is especially low during *Coffea arabica* bean development; *CaXMT1* and *CaDXMT2* expression is drastically reduced compared to what was observed in leaves (Fig. 4b, d). This has a considerable impact on caffeine accumulation, reduced by 50 % at the end of bean maturation. *CaXMT2* and *CaMXMT2* are poorly expressed during coffee bean development in Arabica, partially explaining why *Coffea eugenioides* also accumulates very low levels of caffeine in beans (Campa et al. 2004). In conclusion, XMT1 (*CcXMT1* and *CaXMT1*) and DXMT (*CcDXMT* and *CaDXMT2*) genes play a major role in caffeine accumulation in leaf and bean in Arabica and Robusta. Previous gene expression in leaves or grain was performed and published using classical RT-PCR (Ogawa et al. 2001; Mizuno et al. 2003b; Koshiro et al. 2006). Similarly, major expression was identified for *CaXMT1* and *CaDXMT2* in Arabica even if quantitative analysis was not possible due to the limitations of the classical PCR. Despite the straightforward relationship between specific transcript accumulation and caffeine metabolism, the possibility of post-transcriptional regulation for these key genes XMT and DXMT needs to be considered even if it has not yet been proven.

A major goal of the coffee research programs is to identify and map quantitative trait loci (QTL) involved in cup quality and pathogen resistance and to use this data to apply MAS (Marker Assisted Selection). In parallel, two international sequencing initiatives have been launched to establish the genome sequence of the double haploid *Coffea arabica* and *Coffea canephora* var. *robusta.* In the near future, the resulting catalog of genetic and genomic information will help breeders integrate both analytical tools and release new coffee varieties with higher productivity, resistance and quality. It is probable that genomic locations of QTLs for sensory traits (bitterness) or caffeine content will coincide with the candidate genes characterized in this study, especially with the XMT1 and DXMT genes. The results presented here demonstrate that caffeine modulation content depends on the transcriptional activity monitoring differential expression patterns for the *XMT1* and *DXMT* genes, explaining the different levels of caffeine between Robusta and Arabica. Through this analysis, for the first time, it has been possible to investigate the impact of the co-existence of the two sub-genomes Robusta and Eugenioides in *Coffea arabica* on a specific metabolism. The lowest caffeine content in Arabica is due to reduced transcriptional activity controlling caffeine metabolism on a Robusta sub-genome associated with weak transcriptional activity from the eugenioides sub-genome. The Robusta sub-genome has evolved in the *Coffea arabica* genetic background due to (1) possible evolution of its own transcriptional activity that controls caffeine metabolism or (2) negative impact from the eugenioides sub-genome, or both simultaneously. The data presented here points to interesting targets for modulating the final caffeine content of “green” coffee beans and hence for improving the quality of Robusta and Arabica coffee.

*Author contribution* IP and CP conceived and designed research. CP and GM conducted experiments. CP, SS, LM and ML contributed genomic and phylogenic analysis of the sequences. LB, SM and JH performed biochemical analysis. CP, ML, SS and IP analyzed the data. IP wrote the manuscript. All authors read and approved the manuscript.

## Electronic supplementary material

Below is the link to the electronic supplementary material.

Supplementary Fig. S1 Alignment of the different genomic sequences encoding the N-methyltransferase identified in this article (PPTX 226 kb)

Supplementary Fig. S2 Unrooted maximum likelihood tree based on the alignment of twenty-six N-methyltransferases involved in caffeine metabolism. GenBank accession numbers are as follows: CaXMT1 (AB048793); CcXMT1 (A4GE69); CcXMT1* (JX978518); CaXMT1* (JX978521); CaXMT2* (JX978522); CcMXMT1* (JX978517); CaMXMT1 (AB048794); CaMXMT1* (JX978519); CaMXMT2 (AB084126); CaMXMT2* (JX978520); CaMTL3* (JX978513); CcMTL2* (JX978508); CTS1 (AB034700); CTS2 (AB054841); CaDXMT1* (KF678863); CaDXMT2* (KJ577793); CcDXMT* (JX978516); CaDXMT1 (AB084125); CtCS7 (AB086415); CCS1 (AB086414); CcDXMT (A4GE70); CaMTL2* (JX978527); CcMTL1* (JX978525); CaMTL1 (AB039725); CaMTL2 (AB048792). The sequences marked with an asterisk have been identified in the present article. Clusters I, II, III and IV correspond to XMT, MXMT, DXMT and MTL protein, respectively (PPTX 963 kb)

Supplementary Table S1 Primers and probes used in TaqMan^®^ quantitative RT-PCR assay (DOCX 22 kb)

## Abbreviations

Ca: Coffea arabica
*Cc*: *Coffea canephora* var. *robusta*
Arabica: *Coffea arabica*
Robusta: *Coffea canephora* var. *robusta*
rpl39: Large ribosomal subunit 39 protein
UBQ: Ubiquitin protein
q-RT-PCR: Quantitative real-time polymerase chain reaction
gDNA: Genomic DNA
XMT: Xanthosine-*N*-methyltranferase
MXMT: 7-methylxanthine-*N*-methyltransferase or theobromine synthase
DXMT: 3,7-dimethylxanthine-*N*-methyltranferase or caffeine synthase
MTL: Methyltransferase-like protein
