# Supplementary figures and images for: Differential regulation of caffeine metabolism in Coffeaarabica (Arabica) and Coffea canephora (Robusta)
Source: Planta. 2014 Sep 24;241(1):179–91. doi: 10.1007/s00425-014-2170-7 (PMC4282694; doi:10.1007/s00425-014-2170-7)

## Slide 1
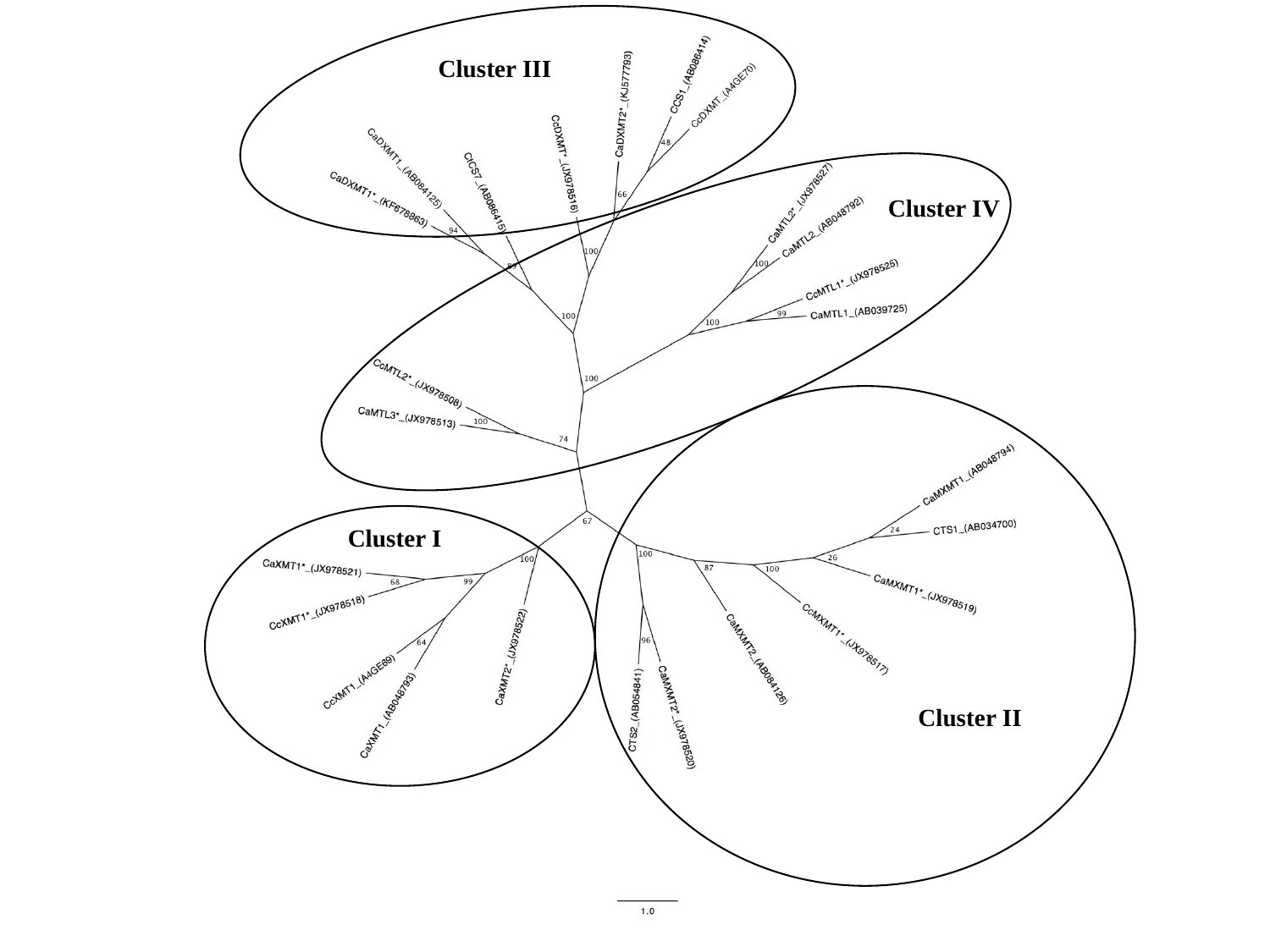

Cluster III
Cluster IV
Cluster I
Cluster II

Supplement: Supplementary file 1 — Supplementary Fig. S1 Alignment of the different genomic sequences encoding the N-methyltransferase identified in this article (PPTX 226 kb) [file 425_2014_2170_MOESM1_ESM.pptx]

## Slide 1
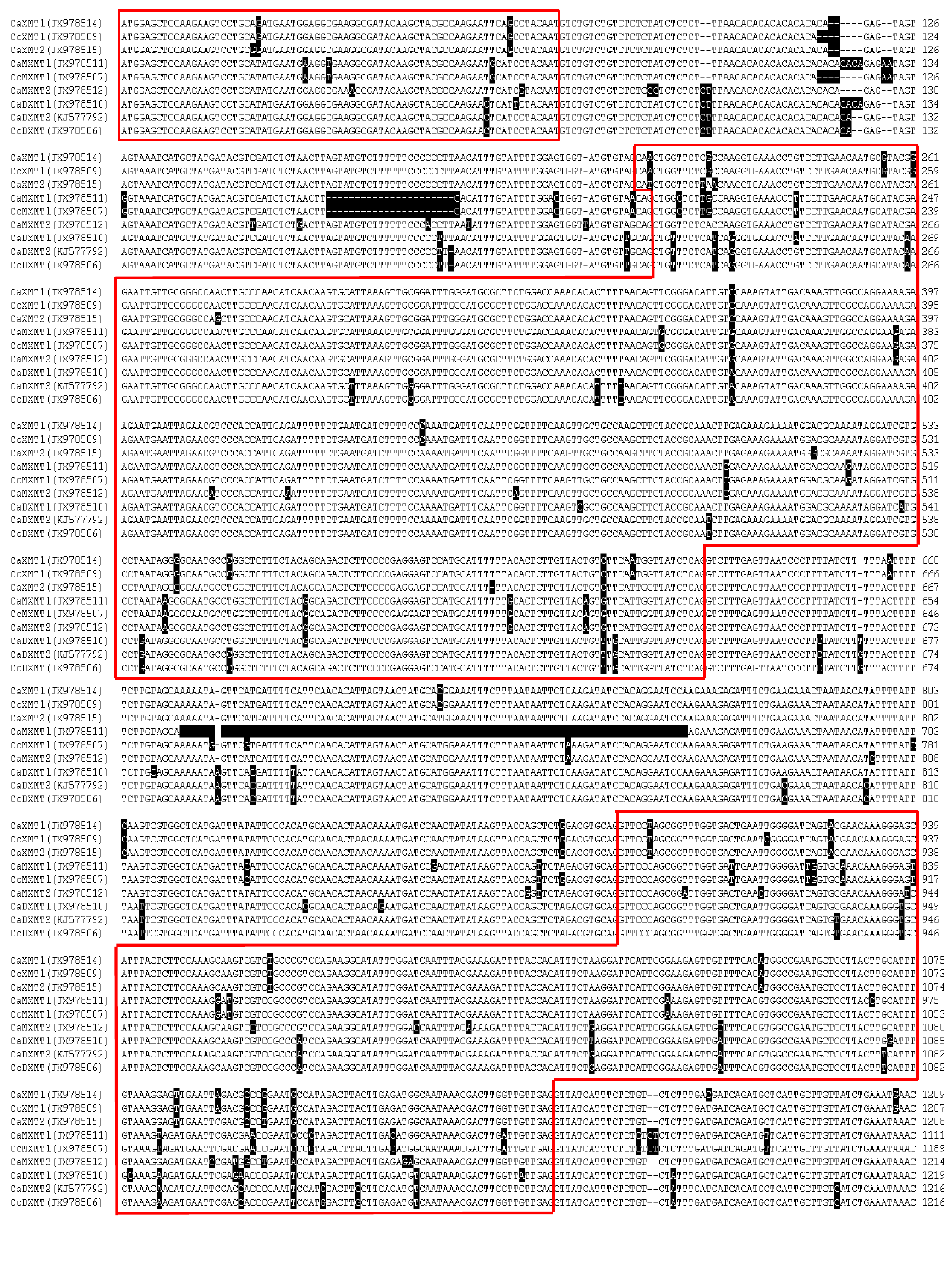

## Slide 2
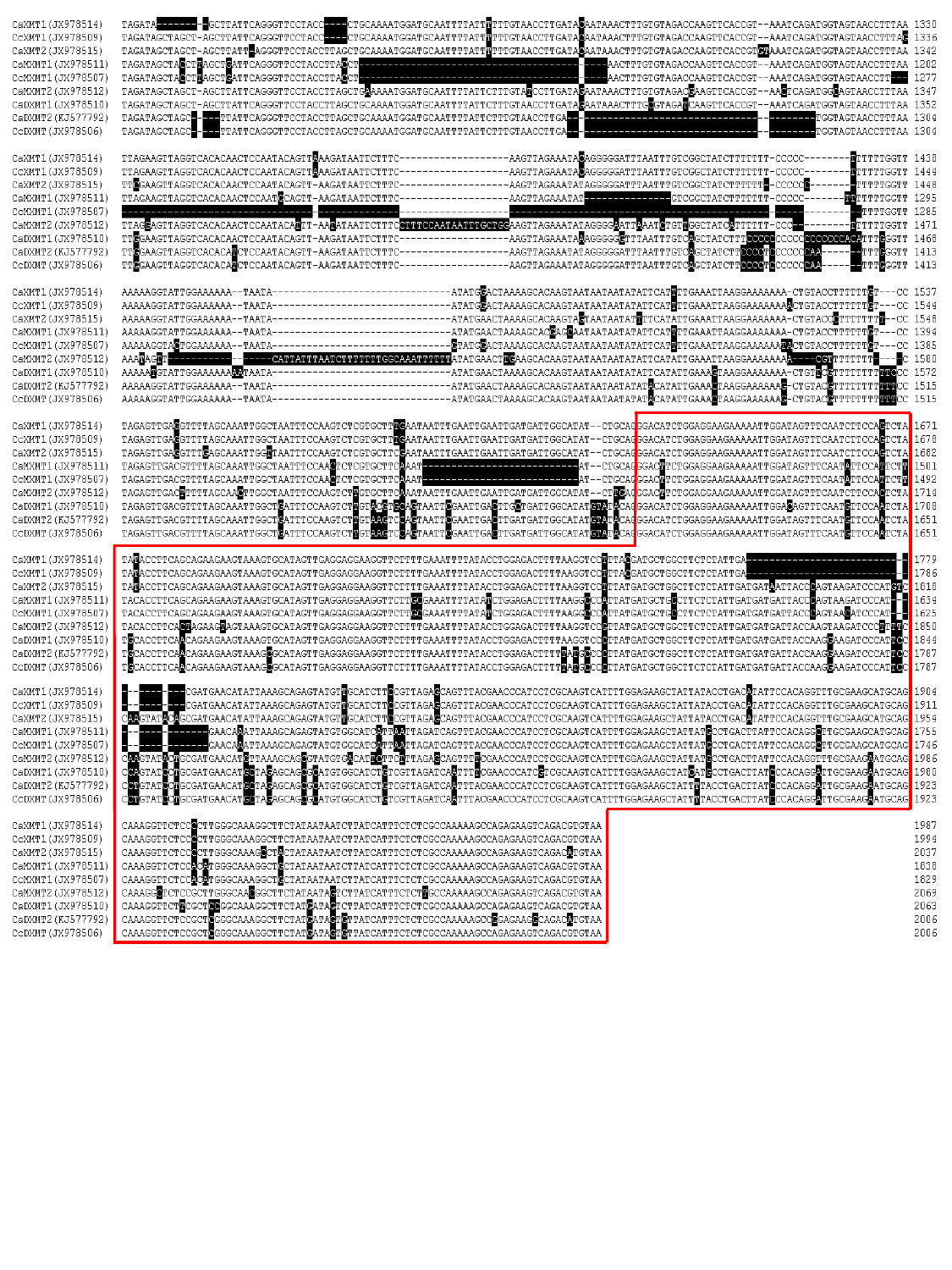

Supplement: Supplementary file 2 — Supplementary Fig. S2 Unrooted maximum likelihood tree based on the alignment of twenty-six N-methyltransferases involved in caffeine metabolism. GenBank accession numbers are as follows: CaXMT1 (AB048793); CcXMT1 (A4GE69); CcXMT1* (JX978518); CaXMT1* (JX978521); CaXMT2* (JX978522); CcMXMT1* (JX978517); CaMXMT1 (AB048794); CaMXMT1* (JX978519); CaMXMT2 (AB084126); CaMXMT2* (JX978520); CaMTL3* (JX978513); CcMTL2* (JX978508); CTS1 (AB034700); CTS2 (AB054841); CaDXMT1* (KF678863); CaDXMT2* (KJ577793); CcDXMT* (JX978516); CaDXMT1 (AB084125); CtCS7 (AB086415); CCS1 (AB086414); CcDXMT (A4GE70); CaMTL2* (JX978527); CcMTL1* (JX978525); CaMTL1 (AB039725); CaMTL2 (AB048792). The sequences marked with an asterisk have been identified in the present article. Clusters I, II, III and IV correspond to XMT, MXMT, DXMT and MTL protein, respectively (PPTX 963 kb) [file 425_2014_2170_MOESM2_ESM.pptx]
