# Supplementary material for: Differential regulation of caffeine metabolism in Coffeaarabica (Arabica) and Coffea canephora (Robusta)
Source: Planta. 2014 Sep 24;241(1):179–91. doi: 10.1007/s00425-014-2170-7 (PMC4282694; doi:10.1007/s00425-014-2170-7)
Supplement: Supplementary file 3 — Supplementary Table S1 Primers and probes used in TaqMan® quantitative RT-PCR assay (DOCX 22 kb) [file 425_2014_2170_MOESM3_ESM.docx]

**Supplementary Table S1** Primers and probes used in TaqMan® quantitative RT-PCR assay. Primers and probes were designed using primer express software (Applied Biosystems)

***gene*** ***sequence***

*CaXMT1* Forward GCTGGCTTCTCTATTGACGATGA

Reverse GCGAGGATGGGTTCGTAAAC

Probe^(1)^ TGCATCTTCCGTTAGAGC

*CcXMT1* Forward TGGCTTCTCTATTGACGATGAACA

Reverse ATGGGTTCGTAAACTGCTCTAACG

Probe^(1)^ AAAGCAGAGTATGTTGCATC

*CaMXMT1* Forward TTACCCAGTAAGATCCCATGAACA

Reverse AGGATGGGTTCGTAAACTGATCTAA

Probe^(1)^ CAGAGTATGTGGCATCAT

*CcMXMT1* Forward TGCTGCCTTCTCTATTGATGATGA

Reverse AGGATGGGTTCGTAAACTGATCTAA

Probe^(1)^ CAGTAAGATCCCATGAACA

*CaXMT2* Forward GATAATTACCCAGTAAGATCCCATGTC

Reverse CTCTAACGGAAGATGCAACATACTCT

Probe^(1)^ AAGTATACAGCGATGAACATA

*CcDXMT* Forward GCATGTGGCATCTGTCGTTAGA

Reverse TGGGATAAGTCAGGTAAAATAGCTTCTC

Probe^(1)^ CCTCGCAAGTCATTTT

*CaDXMT2* Forward GCATGTGGCATCTGTCGTTAGA

Reverse TGGGATAAGTCAGGTAAAATAGCTTCTC

Probe^(1)^ CCTCGCAAGTCATTTT

*rpl39* Forward GAACAGGCCCATCCCTTATTG

Reverse CGGCGCTTGGCAATTGTA

Probe^(2)^ ATGCGCACTGACAACA

*UBQ* Forward TGCTGTACTCTGTTGAAGCTGTCTT

Reverse CCCATGCACGATAACACCAA

Probe^(2)^ TGTTGAACTGAACTTGC

^(1)^ MGB Probes were labelled at the 5’ with fluorescent reporter dye 6-carboxyfluorescein (FAM) and at the 3’

with quencher dye 6-carboxy-tetramethyl-rhodamine (TAMRA). All sequences are given 5’ to 3’. ^(2)^ MGB Probes probe were labelled at the 5’ with fluorescent reporter dye VIC and at the 3’ end with quencher TAMRA
